# Desenvolvimento de protótipo de instrumento para aferir autonomia culinária de mulheres

**DOI:** 10.1590/0102-311XPT078924

**Published:** 2025-03-31

**Authors:** Luciana Maldonado, Mariana Fernandes Brito de Oliveira, Inês Rugani Ribeiro de Castro

**Affiliations:** 1 Instituto de Nutrição, Universidade do Estado do Rio de Janeiro, Rio de Janeiro, Brasil.; 2 Universidade Federal do Rio de Janeiro, Macaé, Brasil.

**Keywords:** Culinária, Alimentação, Protótipo de Prova de Conceito, Cooking, Feeding, Proof of Concept Prototype, Culinaria, Alimentación, Prototipo de Prueba de Concepto

## Abstract

Este estudo objetivou desenvolver o protótipo de um instrumento para avaliar a autonomia culinária de mulheres. Para isso, foram adotados o modelo conceitual de autonomia culinária e o referencial teórico-metodológico de desenvolvimento processual de instrumentos. As etapas realizadas foram: identificação de manifestações empíricas (expressões) do construto de autonomia culinária e elaboração de itens por meio de revisão da literatura, de dois grupos focais (n = 20) e de oficina de especialistas (n = 25); validação de conteúdo e semântica de itens por meio de painel de especialistas (n = 20); avaliação da versão preliminar do protótipo (estrutura dimensional, semântica e gradiente de intensidade dos itens) em oficina com especialista em desenvolvimento de instrumentos; e dois pré-testes (n = 30). A versão final do protótipo do instrumento de autonomia culinária contém 64 itens e é composta por dois blocos: conhecimentos, hábitos e atitudes individuais, com 38 itens; e aspectos do domicílio e práticas dos moradores, com 26 itens. Está em andamento estudo de avaliação da validade e da confiabilidade deste instrumento com mulheres de quatro regiões brasileiras. A aplicação desse instrumento em diferentes realidades poderá subsidiar políticas públicas de alimentação e nutrição, assim como qualificar a atenção nutricional no Sistema Único de Saúde (SUS).

## Abstract (en)

This study aimed to develop a prototype of an instrument to assess women’s culinary autonomy. The conceptual model of culinary autonomy and the theoretical-methodological framework of procedural development of instruments were adopted for this. Steps involved finding empirical manifestations (expressions) of the construct of culinary autonomy and elaboration of items by a literature review, two focus groups (n = 20), a workshop with specialists (n = 25); content and item semantics validation by a panel of experts (n = 20); the evaluation of a preliminary version of the prototype (dimensional structure, semantics, and item intensity gradient) in a workshop with a specialist in instrument development; and two pre-tests (n = 30). The final version of the prototype of the culinary autonomy instrument contains 64 items and two blocks: individual knowledge, habits, and attitudes (38 items) and aspects of the household and residents’ practices (26 items). A study is underway to evaluate the validity and reliability of this instrument with women from four Brazilian regions. Applying this instrument in different realities can support food and nutrition public policies as well as qualify nutritional care in the Brazilian Unified National Health System.

## Abstract (es)

Este estudio tuvo como objetivo desarrollar el prototipo de un instrumento para evaluar la autonomía culinaria de las mujeres. Para ello, se adoptó el modelo conceptual de autonomía culinaria y el marco teórico-metodológico de desarrollo procedimental de instrumentos. Se realizaron las siguientes etapas: identificación de manifestaciones empíricas (expresiones) del constructo de autonomía culinaria y elaboración de ítems mediante revisión de literatura, de dos grupos focales (n = 20) y de talleres de expertos (n = 25); validación de contenido y semántica de ítems mediante un panel de expertos (n = 20); evaluación de la versión preliminar del prototipo (dimensión estructural, semántica y grado de intensidad de los ítems) en un taller con especialista en desarrollo de instrumentos; y dos pretests (n = 30). La versión final del prototipo del instrumento de autonomía culinaria contiene 64 ítems y consta de dos bloques: conocimiento, hábitos y actitudes individuales, con 38 ítems; y aspectos de los hogares y prácticas de los residentes, con 26 ítems. Se está realizando un estudio a mujeres de cuatro regiones brasileñas para evaluar la validez y confiabilidad de este instrumento. La aplicación de este instrumento en diferentes realidades puede apoyar las políticas públicas de alimentación y nutrición, así como cualificar la atención nutricional en el Sistema Único de Salud brasileño.

## Introdução

A valorização da culinária doméstica tem sido reconhecida como iniciativa estratégica para promoção da alimentação adequada e saudável em nível mundial, considerando que essa prática pode produzir impactos positivos para os sistemas alimentares, os valores culturais e sociais envolvidos com a alimentação e para a saúde das populações, por contribuir de diferentes maneiras para a redução de todas as formas de má nutrição ^1^. No entanto, a associação entre culinária, alimentação e saúde ainda apresenta evidências limitadas, apontando a necessidade de mais estudos conceituais e de identificação de determinantes, facilitadores e barreiras para esta prática ^2^.

A culinária doméstica tem sido abordada por meio do desenvolvimento de construtos teóricos e respectivos instrumentos de aferição como *food literacy*, construto oriundo dos estudos sobre *health literacy*, elaborado com base no conhecimento das pessoas sobre planejar, preparar e consumir refeições ^3^ ^,^ ^4^; *cooking skills*, construto estruturado em torno das técnicas de preparo de alimentos ^5^; *food skills*, construto que incorpora às técnicas culinárias as práticas relativas à organização da rotina de alimentação ^5^ ^,^ ^6^ ^,^ ^7^; *healthy cooking*, um modelo conceitual que identifica comportamentos para cozinhar com impacto sobre desfechos de saúde ^8^; e *food agency*, construto que incorpora a agência humana e considera a pessoa como aquela capaz de promover mudanças em seu contexto para manter a prática da culinária doméstica ^9^ ^,^ ^10^. No Brasil, alguns estudos têm realizado adaptação de instrumentos já validados ^11^ ^,^ ^12^.

Em busca de um conceito mais abrangente que incorporasse elementos contextuais e avançasse na concepção sobre as habilidades individuais para cozinhar em casa, sendo coerente com a realidade brasileira, foi desenvolvido o modelo conceitual de autonomia culinária (MCAC). Nele, a autonomia culinária é definida como: “*a capacidade de pensar, decidir e agir para preparar refeições em casa, usando majoritariamente alimentos in natura ou minimamente processados, sob a influência das relações interpessoais, do meio ambiente, dos valores culturais, do acesso a oportunidades e da garantia de direitos*” ^13^ (p. 1). Com base na teoria ecológica de sistemas, o modelo é estruturado em multiníveis: Agente, Microssistema, Mesossistema, Exossistema, Macrossistema e Cronossistema. Neles se distribuem 24 componentes necessários ao desenvolvimento da autonomia culinária, que expressam a interdependência entre habilidades culinárias, agência humana e políticas públicas. O reconhecimento da interação desses múltiplos componentes pretende dar visibilidade à complexidade dessa prática no cotidiano em diferentes arranjos domiciliares. Embasado na abordagem das capacidades humanas, esse conceito porpõe que, para cozinhar em casa diariamente, é necessário, além do domínio de certas técnicas, ter também “*a liberdade para cozinhar de maneira saudável, desenvolvida a partir das habilidades individuais, em um ambiente favorável, ou seja, com recursos disponíveis para a ação do agente*” ^13^ (p. 14), incluindo, ainda, a responsabilidade do Estado nessa importante prática promotora de saúde. Essa complexidade também reflete o recorte conceitual sobre que alimentos são utilizados para cozinhar. Alinhado ao *Guia Alimentar para a População Brasileira*, o modelo considera que a culinária doméstica para promover alimentação adequada e saudável está baseada no preparo de refeições com alimentos *in natura* ou minimamente processados, uma vez que as evidências sobre os riscos à saúde associados ao consumo de alimentos ultraprocessados são cada vez mais robustas ^14^.

Buscando fazer o traslado do plano teórico-conceitual do MCAC ao empírico para viabilizar a aferição do construto de autonomia culinária, o objetivo deste estudo foi desenvolver o protótipo de um instrumento para avaliar a autonomia culinária de mulheres, sendo essa a primeira iniciativa de operacionalização do conceito em um instrumento de aferição. A escolha desse subgrupo populacional se justifica pelo fato de que pesquisas nacionais e internacionais continuam apontando as mulheres ainda como, quase exclusivamente, responsáveis por preparar as refeições em casa ^15^ ^,^ ^16^ ^,^ ^17^.

Diante do desafio de captar a complexidade da culinária doméstica, que vai além da esfera individual, entende-se que a construção de um instrumento para aferir a autonomia culinária poderá contribuir para dimensionar a magnitude desse fenômeno e qualificar as abordagens sobre o tema no âmbito de políticas públicas para a promoção da alimentação adequada e saudável que incluam a valorização e o fortalecimento da prática da culinária doméstica, e também para o apoio ao cuidado em saúde e nutrição em nível individual e/ou familiar.

## Métodos

Adotou-se o referencial teórico-metodológico de desenvolvimento processual de instrumentos multidimensionais, sendo realizadas as etapas descritas a seguir para desenvolvimento do protótipo do instrumento para aferir o construto de autonomia culinária, com base em Reichenheim & Bastos ^18^, Streiner et al. ^19^ e DeVellis ^20^. Por se tratar de procedimentos metodológicos qualitativos, o número de encontros e o número de participantes foi definido de acordo com a intenção de cada etapa e considerando a saturação das questões a serem investigadas com vistas ao aperfeiçoamento do protótipo, sem a pretensão de inferir resultados ^21^.

### Identificação de manifestações empíricas do construto

Foram realizados dois grupos focais ^22^ com mulheres adultas de diferentes níveis socioeconômicos em setembro e dezembro de 2019 na cidade do Rio de Janeiro. O primeiro ocorreu em uma escola da rede municipal de ensino, com a participação de seis mães de crianças de 6 a 10 anos; e o segundo, em uma unidade básica da rede municipal de atenção à saúde, com a participação de 14 mulheres de um grupo de promoção de saúde. As perguntas utilizadas como disparadoras da discussão foram: “Você cozinha em casa as refeições do dia a dia?”, “Que aspectos favorecem essa prática?” e “Que aspectos dificultam essa prática?”. Os grupos focais foram conduzidos pela primeira autora em espaço reservado para a atividade com apoio de duas pesquisadoras que ficaram responsáveis por gravar o encontro, identificar quem falava, registrar gestos e manifestações subjetivas e transcrever as falas. Cada encontro durou em torno de uma hora.

Com base no material empírico levantado, foram realizados pela equipe de pesquisa os seguintes procedimentos: a seleção dos componentes do modelo a serem contemplados no instrumento; a definição de categorias teóricas, que expressam um recorte daquele componente e visam estabelecer uma transição entre os elementos teóricos e suas manifestações empíricas, de forma a cobrir todo o construto de autonomia culinária; e a elaboração de uma ou mais assertivas para cada categoria teórica, construída com base em uma acepção positiva do construto, complementando a sentença “A pessoa tem mais condições/oportunidades para desenvolver autonomia culinária quando/se...”. Nessa fase também houve a postulação preliminar das dimensões do instrumento, propostas com base nos sistemas que compõem o MCAC: dimensão individual; dimensão relacional ou comunitária; e a dimensão de políticas públicas.

### Confecção e seleção de itens

Em março de 2020, de forma remota, realizou-se oficina de trabalho com 15 especialistas que atenderam aos seguintes critérios: ter *expertise* em habilidades culinárias, sistemas e ambientes alimentares, educação alimentar e nutricional e/ou segurança alimentar e nutricional; ter doutorado concluído; e atuar em projetos de pesquisa e extensão em alimentação e nutrição. Visou-se: analisar a abrangência, a pertinência e a suficiência das assertivas e categorias propostas para cobrir o construto de autonomia culinária; identificar lacunas e redundâncias; e apreciar as dimensões propostas para o instrumento. Os especialistas deveriam comentar sobre a coerência entre os componentes e suas respectivas categorias teóricas e assertivas; e responder, por meio de uma escala Likert de cinco pontos para graus de concordância, as seguintes perguntas sobre cada assertiva: “É relevante para o conceito de autonomia culinária?”, “É viável/de fácil aferição?” e “Apresentará resistência para ser respondida?”.

### Validação de conteúdo dos itens

Com base nos resultados da oficina, foi elaborada a primeira versão dos itens do instrumento. Essa foi então submetida à avaliação de um painel de especialistas, de forma remota e assíncrona, com o objetivo de validar o conteúdo dos itens elaborados até aquele momento ^19^. Dos 29 especialistas convidados, participaram 20, sendo 10 já consultados na oficina anterior. Os 10 novos especialistas atenderam aos seguintes critérios de seleção: ter *expertise* na construção de instrumentos em áreas temáticas afins à autonomia culinária e em estudos qualitativos sobre alimentação adequada e saudável; ter doutorado concluído; e atuar em projetos de pesquisa e extensão em alimentação e nutrição. Além da análise semântica de cada item, os participantes foram convidados a responder sobre a relevância, a pertinência e a clareza de cada item utilizando uma escala Likert de quatro pontos sobre graus de concordância variando de “discordo fortemente (1)” a “concordo fortemente (4)”. A validação de conteúdo dos itens foi realizada por meio do cálculo da *content validity ratio* (CVR), considerando-se o ponto de corte de 0,62 para descarte dos itens.

### Pré-teste I

O objetivo desta etapa foi analisar a adequação da primeira versão do protótipo quanto a: domínio populacional (mulheres adultas de diferentes raças e níveis de escolaridade); modo de aplicação (entrevista face a face); cenários de administração (domicílios, unidades básicas de saúde ou outros equipamentos de políticas públicas); aspectos relativos à adequação semântica e de opções de resposta dos itens; e aspectos como aceitação e impacto emocional. A primeira jornada de pré-teste foi realizada em fevereiro de 2021, por meio de entrevistas face a face com 10 mulheres socialmente vulneráveis que participavam de uma organização não governamental.

Após a realização dessa jornada de pré-teste, a equipe de pesquisa realizou os ajustes necessários: alteração em enunciados e/ou opções de respostas; inclusão ou exclusão de itens; e reorganização do encadeamento de itens.

### Oficina com especialista em desenvolvimento de instrumentos

Essa oficina ocorreu por meio de três reuniões remotas, em abril de 2021, envolvendo um pesquisador de referência em desenvolvimento de instrumentos e a equipe de pesquisa. Nela foram abordados a estrutura dimensional do instrumento e a pertinência da formulação de um escore, a definição dos componentes do MCAC a serem incluídos no instrumento, o desenvolvimento do mapa de construto e a elaboração de itens. Após a consolidação de todas as contribuições, a segunda versão do protótipo foi elaborada e submetida a nova jornada de pré-teste.

### Pré-teste II

A segunda jornada de pré-teste aconteceu em julho de 2021 com dois grupos de mulheres de diferentes níveis socioeconômicos moradoras da cidade do Rio de Janeiro: 10 trabalhadoras da empresa de serviço de limpeza de uma universidade, entrevistadas face a face (que trabalharam presencialmente durante a pandemia de COVID-19); e 10 mulheres de classe média, mães de crianças de 6 a 10 anos matriculadas em uma escola da rede privada, entrevistadas remotamente (em função da suspensão das aulas durante a pandemia). Assim como na primeira jornada, foram realizados os ajustes necessários.

### Aspectos éticos

O estudo foi cadastrado no Comitê de Ética da Universidade do Estado do Rio de Janeiro/Hospital Universitário Pedro Ernesto (CAAE: 78647017.2.3001.5259 e aprovado de acordo com parecer nº 2.611.948). Foram incluídos especialistas e mulheres que concordaram em participar do estudo e assinaram Termo de Consentimento Livre e Esclarecido.

## Resultados

### Do MCAC à elaboração de itens para o protótipo do *Instrumento de Autonomia Culinária* (IAC)

Nos grupos focais, as falas indicaram manifestações empíricas referentes aos diferentes componentes do MCAC, a saber: cozinhavam diariamente, com exceção de algumas que moravam sozinhas; utilizavam alimentos *in natura* ou minimamente processados para preparar as refeições e alimentos ultraprocessados em lanches, principalmente para as crianças; não costumavam congelar alimentos, pois consideravam que essa técnica alterava a qualidade nutricional e sensorial dos alimentos; apresentavam dificuldades para planejar refeições para mais de um dia; sentiam-se sobrecarregadas com o preparo de refeições, em especial por ser um trabalho diário e que requer atenção, o que torna difícil cozinhar e fazer outras tarefas da casa ao mesmo tempo; consideravam que cozinhar em casa é uma estratégia para economizar dinheiro, sendo necessária para que o orçamento da família seja suficiente para cobrir todas as despesas; gostariam de uma cozinha maior, principalmente para dividir as tarefas de preparo das refeições e arrumação da cozinha; apontaram preocupação com o preço do gás e desconfiança em relação à propaganda de alimentos e à presença de agrotóxicos nos alimentos consumidos; relataram que o grupo familiar comia reunido, em geral, assistindo à televisão; mencionaram ir com baixa frequência a feiras livres para compra de alimentos, em função do horário de funcionamento ou da distância até a casa; relataram que o comércio perto de casa costumava ser mais caro; e reconheceram a necessidade de mobilização comunitária para demandar políticas públicas, por exemplo, para regularizar o abastecimento de água encanada.

Em um momento inicial, todos os componentes do modelo foram incluídos no protótipo, sendo especificados em categorias teóricas e estas em assertivas (Quadro 1), e submetidas à avaliação de especialistas.

**Quadro 1:** Traslado do modelo conceitual para manifestações empíricas de autonomia culinária. Brasil, 2021.

COMPONENTES DO MODELO CONCEITUAL DE AUTONOMIA CULINÁRIA	CATEGORIA TEÓRICA	ASSERTIVA SOBRE O CONSTRUTO (MANIFESTAÇÃO EMPÍRICA) A PESSOA TEM MAIS CONDIÇÕES/OPORTUNIDADES PARA DESENVOLVER AUTONOMIA CULINÁRIA QUANDO/SE...
NÍVEL AGENTE		
Conhecimento	Grupos de alimentos de acordo com a extensão e o propósito de processamento	Sabe que alimentos *in natura* ou minimamente processados devem compor as refeições diariamente
		Sabe que alimentos ultraprocessados devem ser evitados
	Variedade de alimentos	Sabe que as refeições devem ser compostas por alimentos variados de cada grupo de alimentos
	Armazenamento de alimentos	Reconhece o congelamento de alimentos como uma boa prática de armazenamento
	Boas práticas de manipulação de alimentos	Sabe que sobras de alimentos devem ser mantidos sob refrigeração para serem consumidos em outras refeições
Interesse para cozinhar	Cozinhar como prática prazerosa	Sente satisfação em cozinhar no cotidiano
Confiança para cozinhar	Utilização de receitas	Confia na sua capacidade para cozinhar, seguindo ou não alguma receita
Disposição para cozinhar	Disposição para cozinhar no dia a dia	Apresenta energia física e mental para cozinhar no cotidiano
Habilidades culinárias	Planejamento de refeições	Realiza planejamento de compras de alimentos para cozinhar
		Realiza planejamento de cardápios
	Criatividade	Prepara refeições com os alimentos disponíveis na geladeira e/ou na despensa sem seguir receitas
	Uso de técnicas mecânicas	Domina técnicas básicas de pré-preparo e preparo de alimentos básicos
	Uso dos sentidos	Faz uso dos sentidos para avaliar os alimentos enquanto cozinha
Atitude estratégica	Otimização dos processos e etapas necessários para cozinhar	Adota práticas para buscar mais praticidade no preparo das refeições
	Organização do tempo para cozinhar	Consegue preservar tempo para preparar as refeições do dia a dia
Experiência e vivência em culinária	Repertório de preparações	Cozinha ou já cozinhou diferentes preparações com alimentos básicos
	Influência da família	Acumulou experiências ou vivências no preparo de alimentos junto a familiares e/ou amigos
NÍVEL MICROSSISTEMA		
Cozinha com infraestrutura básica	Estrutura física	A cozinha do domicílio possui estrutura minimamente suficiente para as atividades culinárias diárias (em relação à espaço físico, espaço para guardar utensílios e alimentos etc.)
	Equipamentos	A cozinha do domicílio possui fogão em funcionamento
		A cozinha do domicílio possui geladeira em funcionamento
	Acesso a água	A cozinha do domicílio possui abastecimento regular de água
	Acesso a gás	A cozinha do domicílio possui abastecimento regular de gás
	Acesso a energia elétrica	A cozinha do domicílio possui abastecimento regular de energia elétrica
Importância atribuída à culinária	Preparo de refeições em casa	Convive no domicílio com pessoas que privilegiam o preparo de refeições em casa em detrimento da compra de alimentos prontos para consumo
	Tradições familiares ou regionais	Convive no domicílio com pessoas que valorizam o preparo de receitas tradicionais da família
	Comensalidade	Convive no domicílio com pessoas que que valorizam a comensalidade
Compartilhamento das atividades culinárias	Divisão de tarefas domésticas	Mora em um domicílio em que há divisão de tarefas domésticas entre as pessoas da casa
	Divisão de tarefas de culinária	Mora em um domicílio em que há divisão de tarefas relacionadas a cozinhar em casa (desde as compras até a limpeza da cozinha) entre as pessoas da casa
	Decisão sobre compras de alimentos	Compartilha a decisão sobre que alimentos serão comprados para preparar as refeições
Poder de compra com análise crítica	Tipo de alimento	Convive no domicílio com pessoas que evitam a compra alimentos com impacto negativo sobre a saúde e/ou sobre o ambiente
	Propaganda de alimentos	Reconhece que as propagandas de alimentos não são uma fonte confiável de informações sobre alimentação
	Origem dos alimentos	Convive no domicílio com pessoas que privilegiam a compra de alimentos diretor do produtor
	Preço dos alimentos	Convive no domicílio com pessoas que monitoram o preço dos alimentos
	Rótulos dos alimentos	Convive no domicílio com pessoas que utilizam o rótulo para tomar decisões sobre a compra de alimentos
NÍVEL MESOSSISTEMA		
Disponibilidade e acesso a alimentos *in natura* ou minimamente processados	Disponibilidade de alimentos em local próximo à moradia	Mora em um local onde alimentos *in natura* e minimamente processados são encontrados a preços acessíveis, com qualidade e com variedade
		Mora em um domicílio ou em uma vizinhança que possui horta doméstica e/ou criação de animais
Movimentos sociais	Ações de *advocacy*	Participa de iniciativas coletivas para incidir sobre políticas públicas para garantia de segurança alimentar e nutricional e promoção da alimentação adequada e saudável
		Participa ou apoia movimentos e políticas feministas
NÍVEL EXOSSISTEMA		
Uso saudável do tempo	Tempo dedicado ao trabalho remunerado	Considera que o tempo dedicado ao trabalho (deslocamento entre a casa e o trabalho e a jornada de trabalho em si) não prejudica as tarefas relacionadas ao preparo das refeições
Seguridade social	Acesso a direitos trabalhistas	Possui um emprego/ocupação com direitos trabalhistas garantidos
	Acesso a programas de proteção social	Em condição de vulnerabilidade, tem acesso a programas sociais de transferência de renda (dinheiro, auxílio gás etc.) ou de distribuição de alimentos
Segurança alimentar e nutricional	Acesso a renda	Tem acesso regular a renda suficiente para preparar comida em casa (alimentos e outros gastos)
	Acesso a informação	Consegue identificar alimentos com alto teor de açúcar, gordura ou sódio ou com edulcorantes com as informações disponíveis no rótulo dos alimentos
		Tem acesso à informação sobre alimentação adequada e saudável em serviços de saúde, na escola da comunidade ou em outros serviços, como os de assistência social
Incentivo à culinária	Ações de educação alimentar e nutricional	Vivencia, por meio de oficinas de culinária, e/ou é incentivada a cozinhar em casa (por meio do atendimento, práticas educativas ou distribuição de impressos) em serviços de saúde, na escola da comunidade ou em outros serviços, como os de assistência social
Incentivo à agricultura familiar e urbana de base agroecológica	Acesso a pontos de venda	Tem acesso a iniciativas/espaços de comercialização (propriedade do agricultor, feira, galpão, lojas) de alimentos da agricultura familiar
NÍVEL MACROSSISTEMA		
Garantia do direito humano à alimentação adequada e saudável	Acesso à alimentação como direito humano	Tem acesso a, pelo menos, três refeições diárias de forma digna sem ter que abrir mão de outras necessidades básicas (preparando em casa, comprando ou acessando alguma política pública como PNAE; PAT; restaurante popular etc.)
Garantia da saúde	Acesso à rede de atenção à saúde como direito	Tem/teve acesso ao serviço público de saúde quando procura
	Igualdade racial	Tem acesso ao cuidado em saúde que respeita as questões raciais (não sofreu maus tratos por ser negra)
Garantia da educação	Acesso à educação como direito	Não precisou parar de estudar para trabalhar (doméstico ou fora de casa)

PAT: Programa de Alimentação do Trabalhador; PNAE: Programa Nacional de Alimentação Escolar.

Fonte: elaboração própria.

Os principais resultados dessa oficina foram: (a) exclusão do componente “valorização da culinária e da alimentação na cultura alimentar”, por considerar que suas manifestações empíricas já estariam presentes em outros componentes; (b) ajustes de algumas assertivas para pessoas que moram sozinhas; (c) confirmação de que as mulheres seriam o público-alvo do instrumento; (d) necessidade de revisão da categoria teórica “preconceito” em função do racismo estrutural presente no contexto brasileiro.

Após essas etapas, foi elaborada uma versão preliminar dos itens a serem incluídos no protótipo do IAC.

### Validação de conteúdo dos itens

No painel de especialistas que validou a primeira versão dos itens, os aspectos mais problematizados foram: (a) categorias teóricas de grupos de alimentos de acordo com a extensão e o propósito de processamento, disposição cotidiana para cozinhar e planejamento de refeições; (b) necessidade de adaptação dos itens para as mulheres que moram sozinhas; (c) inclusão de itens para captar a sobrecarga das mulheres com o trabalho doméstico, incluindo a culinária; (d) problematização sobre os itens relativos aos componentes de políticas públicas, considerando que estes seriam somente determinantes da autonomia culinária; (e) diferentes estruturas de opções de resposta; (f) quantidade de itens do instrumento; (g) e se o tipo de alimento preparado pela pessoa realmente teria impacto no gradiente de autonomia culinária.

Os especialistas identificaram lacunas sobre: componentes dos sistemas alimentares; aspectos simbólicos do cozinhar, incluindo uso do tempo e prazer; compra de alimentos em função de preços, impactos ambientais e na saúde e distância do estabelecimento.

As pontuações quanto à relevância, pertinência e clareza foram consolidadas e o valor do CVR para cada item variou de 0,67 a 1,0 (Quadros 2, 3 e 4). Ao final dessas etapas, elaborou-se a primeira versão do protótipo que foi submetida a pré-teste com o público-alvo do instrumento.

**Quadro 2:** Validação de conteúdo dos itens do protótipo e mudanças neles realizadas a cada etapa do estudo - níveis Agente e Microssistema do modelo conceitual de autonomia vulinária. Brasil, 2021.

ITEM	CVR *			MUDANÇA APÓS:			
	R	P	C	PAINEL	PRÉ-TESTE I	OFICINA COM ESPECIALISTA	PRÉ-TESTE II
CONHECIMENTO							
1. Os alimentos básicos, como arroz, feijão, legumes, verduras, frutas, ovos e carnes, devem estar presentes nas refeições diariamente	1,00	1,00	1,00	Alteração no enunciado e na opção de resposta	Alteração na opção de resposta	Itens aglutinados; alteração no enunciado e na opção de resposta	Sem alteração
2. Os alimentos como refrigerantes, salsicha, macarrão instantâneo, biscoitos, guloseimas etc., devem ser evitados na alimentação do dia a dia	1,00	1,00	0,90	Alteração no enunciado e na opção de resposta	Alteração na opção de resposta		
3. Quanto maior a variedade de alimentos (diferentes tipos de grãos, legumes, verduras, frutas e carnes) utilizados para preparar as refeições, melhor	0,89	1,00	0,79	Alteração no enunciado e na opção de resposta	Alteração na opção de resposta	Alteração no enunciado e na opção de resposta	Sem alteração
4. O congelamento é uma forma de fazer os alimentos durarem por mais tempo	0,89	1,00	1,00	Alteração no enunciado e na opção de resposta	Alteração na opção de resposta	Alteração no enunciado e na opção de resposta	Alteração no enunciado
5. As sobras de comida que serão aproveitadas em outras refeições devem ser guardadas na geladeira	0,89	1,00	1,00	Alteração no enunciado e na opção de resposta	Alteração na opção de resposta	Alteração no enunciado e na opção de resposta	Alteração no enunciado
INTERESSE PARA COZINHAR							
6. Você se interessa em cozinhar usando novos ingredientes ou testando formas diferentes de preparar os alimentos?	1,00	1,00	0,79	Alteração da categoria teórica	Alteração no enunciado	Item subdividido em 3 por gradiente de intensidade	1. Sem alteração 2. Pequena alteração no enunciado 3. Alteração no enunciado
CONFIANÇA PARA COZINHAR							
7. Você se sente confiante para preparar refeições utilizando, principalmente, alimentos básicos, por exemplo: arroz, feijão, legumes, verduras, carnes e ovos?	1,00	1,00	0,90	Sem alteração	Sem alteração	Excluído	-
8. Você se sente confiante para preparar uma comida que você nunca fez seguindo uma receita?	1,00	1,00	1,00	Pequena alteração no enunciado	Alteração no enunciado	Item subdividido em 2 por gradiente de intensidade	Alteração de enunciado em 2 itens
9. Você se sente confiante para refazer um prato ou preparação culinária que não tenha ficado boa em tentativas anteriores?	0,70	0,90	0,90	Alteração no enunciado	Alteração no enunciado	Sem alteração	Sem alteração
DISPOSIÇÃO PARA COZINHAR							
10. Você tem disposição para cozinhar no dia a dia?	0,90	0,90	0,70	Alteração no enunciado	Sem alteração	Item subdividido em 3 por gradiente de intensidade	Alteração na ordem do questionário
HABILIDADE CULINÁRIAS							
11. Você se organiza para não ficar sem os alimentos básicos em casa (por exemplo, anota o que precisa comprar; faz lista de compras; combina quem vai comprar, quando e onde comprar)?	1,00	1,00	0,70	Alteração no enunciado	Sem alteração	Alteração no enunciado; retirada de exemplos; inclusão de um item por gradiente de intensidade	Suspensão do item incluído
12. Você planeja as preparações culinárias ou pratos que vão compor suas refeições para combinar diferentes alimentos e variar no decorrer da semana?	0,70	1,00	0,80	Alteração no enunciado	Sem alteração	Alteração no enunciado	Sem alteração
13. Você faz adaptações em receitas, preparações culinárias ou pratos?	0,89	1,00	0,89	Excluído temporariamente	-	Item reincluído com alteração no enunciado	Alteração no enunciado
14. Você prepara refeições com os alimentos que estão disponíveis em casa sem seguir nenhuma receita?	0,90	0,80	0,90	Alteração no enunciado	Sem alteração	Alteração no enunciado	Sem alteração
15. Você acha que sabe o básico para cozinhar em casa (por exemplo, limpar, picar, temperar e cozinhar os alimentos)?	1,00	1,00	0,80	Pequena alteração no enunciado	Sem alteração	Itens aglutinados e reorganizados em 5 itens	Alteração de enunciado em 2 itens
16. Você sabe preparar feijão?	0,79	0,79	0,89	Excluído temporariamente	-		
17. Você tempera os alimentos usando temperos naturais, por exemplo: alho, cebola, salsinha, colorau, orégano, coentro, pimenta...?	0,90	0,90	1,00	Excluído temporariamente	-		
18. Você utiliza o olfato (cheiro), a visão, o tato ou o paladar (gosto) para conferir se a comida está boa (por exemplo: o cheiro do bolo quando está pronto; a cor da carne enquanto cozinha; o gosto da comida para provar o tempero)?	1,00	1,00	0,89	Alteração no enunciado	Sem alteração	Item subdividido em 4 por gradiente de intensidade	Sem alteração
ATITUDE ESTRATÉGICA							
19. Você se organiza para deixar comida pronta para mais de uma refeição?	0,89	0,89	0,78	Alteração no enunciado	Sem alteração	Item subdividido em 2 por gradiente de intensidade	Sem alteração
20. Você costuma adiantar o preparo das refeições mantendo alguns alimentos quase prontos na geladeira, por exemplo: folhas lavadas, feijão cozido, carnes temperadas, temperos picados, molhos caseiros?	1,00	1,00	0,89	Alteração no enunciado	Alteração no enunciado	Alteração no formato do item; retirada de exemplos	Subdivisão em 2 itens por gradiente de intensidade
21. Você costuma preparar mais de um alimento ao mesmo tempo para economizar tempo, por exemplo: pica alguns alimentos enquanto cozinha outros?	0,80	1,00	1,00	Excluído temporariamente	-	Revisão da categoria teórica; retirada de exemplos; subdivisão em 2 itens por gradiente de intensidade	1. Alteração no enunciado 2. Sem alteração
EXPERIÊNCIA E VIVÊNCIA EM CULINÁRIA							
22. Você cozinha ou já cozinhou diferentes preparações ou pratos com alimentos básicos, por exemplo: arroz, feijão, legumes, verduras, carnes, ovos, temperos naturais?	0,89	0,89	0,68	Alteração no enunciado	Sem alteração	Subdivisão em 3 itens por gradiente de intensidade; retirada de exemplos	Alteração de enunciado em 3 itens
23. Você aprendeu a cozinhar com pessoas da família ou com pessoas próximas?	1,00	1,00	0,89	Sem alteração	Sem alteração	Subdivisão em 3 itens por gradiente de intensidade	Alteração de enunciado em 1 item

C: clareza; CVR: *content validity ratio*; P: pertinência; R: relevância.

Fonte: elaboração própria.

* CVR = (número de especialistas que classificaram cada item como “relevante” [4 ou 3, onde 4 = “altamente relevante”; 3 = “bastante relevante” ou “altamente relevante”, mas precisa ser reescrito] - número total de respondentes/2)/número total de respondentes/2. O mesmo se aplica para “pertinente” e “claro”.

**Quadro 3:** Validação de conteúdo dos itens do protótipo e mudanças neles realizadas a cada etapa do estudo - nível Mesossistema do modelo conceitual de autonomia culinária. Brasil, 2021.

ITEM	CVR *			MUDANÇA APÓS:			
	R	P	C	PAINEL	PRÉ-TESTE I	OFICINA COM ESPECIALISTA	PRÉ-TESTE II
COZINHA COM INFRAESTRUTURA BÁSICA							
24. Onde você mora, a cozinha tem espaço suficiente para as atividades relacionadas ao preparo das refeições (cozinhar, guardar alimentos e guardar panelas, pratos e talheres)?	1,00	1,00	0,89	Pequena alteração no enunciado	Sem alteração	Subdivisão em 2 itens	Sem alteração
25. Onde você mora, a cozinha tem fogão funcionando?	1,00	1,00	1,00	Pequena alteração no enunciado	Sem alteração	Sem alteração	Sem alteração
26. Onde você mora, a cozinha tem geladeira funcionando?	1,00	1,00	1,00	Pequena alteração no enunciado	Sem alteração	Sem alteração	Sem alteração
27. Nos últimos três meses, faltou água na sua casa?	1,00	1,00	1,00	Pequena alteração no enunciado	Sem alteração	Sem alteração	Sem alteração
28. Nos últimos três meses, faltou gás na sua casa?	1,00	1,00	1,00	Pequena alteração no enunciado	Sem alteração	Sem alteração	Sem alteração
29. Nos últimos três meses, faltou luz na sua casa?	1,00	1,00	1,00	Pequena alteração no enunciado	Sem alteração	Sem alteração	Sem alteração
IMPORTÂNCIA ATRIBUÍDA À CULINÁRIA							
30. Você mora com pessoas que acham melhor cozinhar em casa do que comprar comida pronta?	1,00	1,00	0,89	Alteração no enunciado; inclusão de opção de resposta	Sem alteração	Alocação em bloco específico	Sem alteração
31. Você mora com pessoas que dão valor a quem cozinha em casa?	0,90	0,90	0,90	Excluído	-	-	-
32. Você mora com pessoas que dão valor a preparar receitas tradicionais da família?	1,00	1,00	1,00	Inclusão de opção de resposta	Pequena alteração no enunciado	Alocação em bloco específico	Sem alteração
33. Você mora com pessoas que dão valor a comer juntas, pelo menos uma vez ao dia?	1,00	1,00	1,00	Inclusão de opção de resposta	Sem alteração	Alocação em bloco específico	Sem alteração
COMPARTILHAMENTO DAS ATIVIDADES CULINÁRIAS							
34. Na sua casa, as tarefas de cuidado da casa são divididas com mais alguém?	1,00	1,00	0,90	Alteração no enunciado e opções de resposta	Alteração no enunciado	Alteração de enunciado e opções de resposta	Sem alteração
35. Na sua casa, as tarefas de preparo das refeições, por exemplo: fazer as compras, preparar a comida, limpar a cozinha, tirar o lixo... são divididas com mais alguém?	1,00	1,00	0,80	Alteração no enunciado e opções de resposta	Alteração no enunciado	Subdivisão em 3 itens por gradiente de intensidade; alteração das opções de resposta	Sem alteração
36. Você decide ou participa da decisão de quais alimentos serão comprados para preparar as refeições?	0,70	0,89	0,90	Alteração no enunciado e opções de resposta	Sem alteração	Alteração de enunciado e opções de resposta	Sem alteração
37. Você ou as pessoas que compram alimentos para sua casa dão preferência a alimentos básicos como arroz, feijão, carnes, ovos, leite, frutas, legumes e verduras?	1,00	1,00	1,00	Pequena alteração no enunciado	Sem alteração	Revisão das categorias teóricas; inclusão de 6 itens por gradiente de intensidade	1. Sem alteração 2. Alteração no enunciado 3. Suspenso 4. Suspenso 5. Sem alteração 6. Sem alteração
38. Você ou as pessoas que compram alimentos para sua casa dão preferência a alimentos orgânicos ou agroecológicos?	0,90	1,00	0,90	Excluído temporariamente	-		
39. Você ou as pessoas que compram alimentos para sua casa dão preferência a comprar alimentos direto dos produtores?	0,79	0,89	0,89	Excluído temporariamente	-		
40. Você ou as pessoas que compram alimentos para sua casa dão preferência a comprar alimentos em locais que vendem principalmente alimentos básicos como feiras livres, feira de produtores, mercearias, sacolão...?	1,00	1,00	1,00	Pequena alteração no enunciado	Excluído temporariamente		
41. Você confia nas informações que aparecem em propagandas de alimentos (no rádio, na TV ou em mídias sociais)?	0,80	0,90	0,80	Sem alteração	Alteração de enunciado	Excluído	-
**DISPONIBILIDADE E ACESSO A ALIMENTOS *IN NATURA* OU MINIMAMENTE PROCESSADOS**							
42. Pensando em alimentos básicos como arroz, feijão, carnes, ovos, leite, frutas, legumes e verduras; onde você mora, consegue comprar esses alimentos com bom preço?	1,00	1,00	0,90	Alteração no enunciado	Sem alteração	Alteração de enunciado opção de resposta	Subdivisão em 2 itens por gradiente de intensidade
43. Pensando em alimentos básicos como arroz, feijão, carnes, ovos, leite, frutas, legumes e verduras; onde você mora, consegue comprar esses alimentos com qualidade?	1,00	1,00	1,00	Alteração no enunciado	Sem alteração	Alteração de enunciado opção de resposta	Subdivisão em 2 itens por gradiente de intensidade
44. Pensando em alimentos básicos como arroz, feijão, carnes, ovos, leite, frutas, legumes e verduras; onde você mora, consegue comprar esses alimentos com variedade?	0,89	0,89	1,00	Alteração no enunciado	Sem alteração	Alteração de enunciado e opção de resposta	Subdivisão em 2 itens por gradiente de intensidade
45. Você costuma utilizar alimentos de horta ou de criações caseiras para preparar as refeições?	0,89	0,89	0,79	Alteração no enunciado	Alteração no enunciado	Alteração de enunciado e opção de resposta	Sem alteração
MOVIMENTOS SOCIAIS							
46. Você participa ou já participou de algum grupo para fazer compras coletivas de alimentos direto do produtor?	0,90	1,00	1,00	Excluído temporariamente	-	Excluído	-
47. Você participa ou já participou de uma horta comunitária?	1,00	1,00	1,00	Excluído temporariamente	-	Excluído	-
48. Você participa ou já participou de iniciativas ou grupos que atuam para reivindicar por alimentação mais saudável, por exemplo: associação de pais da escola, Conselho de Alimentação Escolar; grupos de ativistas em defesa de regras para propagandas de alimentos para crianças?	0,80	0,90	0,90	Alteração no enunciado	Sem alteração	Excluído	-
49. Você já participou ou apoiou grupos ou movimentos que defendem os direitos das mulheres?	0,78	0,78	0,89	Alteração no enunciado	Sem alteração	Excluído	-

C: clareza; CVR: *content validity ratio*; P: pertinência; R: relevância.

Fonte: elaboração própria.

* CVR = (número de especialistas que classificaram cada item como “relevante” [4 ou 3, onde 4 = “altamente relevante”; 3 = “bastante relevante” ou “altamente relevante”, mas precisa ser reescrito] - número total de respondentes/2)/número total de respondentes/2. O mesmo se aplica para “pertinente” e “claro”.

**Quadro 4:** Validação de conteúdo dos itens do protótipo e mudanças neles realizadas a cada etapa do estudo - níveis Exossistema e Macrossistema do modelo conceitual de autonomia culinária. Brasil, 2021.

ITEM	CVR *			MUDANÇA APÓS:			
	R	P	C	PAINEL	PRÉ-TESTE I	OFICINA COM ESPECIALISTA	PRÉ-TESTE II
USO SAUDÁVEL DO TEMPO						
50. Você considera que o tempo dedicado diariamente ao seu trabalho prejudica o preparo das refeições em casa (considere a jornada de trabalho e o tempo gasto no deslocamento entre a casa e o trabalho)?	1,00	1,00	1,00	Pequena alteração no enunciado	Sem alteração	Excluído	-
SEGURIDADE SOCIAL						
51. Você ou outro morador da sua casa tem um emprego com direitos trabalhistas garantidos, por exemplo: carteira assinada, férias, 13º salário ou Fundo de Garantia por Tempo de Serviço?	0,79	0,89	1,00	Alteração no enunciado	Sem alteração	Excluído	-
52. Nos últimos três meses, os alimentos acabaram antes que você ou outros moradores da sua casa tivessem dinheiro para comprar mais comida?	1,00	1,00	1,00	Sem alteração	Sem alteração	Excluído	-
53. Você ou outro morador da sua casa recebe algum benefício do Governo para complementar sua renda, por exemplo: auxílio em dinheiro, auxílio gás (não considere aposentadoria)?	0,89	1,00	1,00	Alteração no enunciado	Sem alteração	Excluído	-
SEGURANÇA ALIMENTAR E NUTRICIONAL						
54. Você recebe alimentos de algum programa do Governo, por exemplo: leite, cesta básica...?	1,00	1,00	1,00	Alteração no enunciado	Alteração no enunciado	Excluído	-
55. Você consegue identificar se um alimento tem muito açúcar, gordura ou sódio com as informações disponíveis no rótulo dos alimentos?	1,00	1,00	0,79	Alteração no enunciado	Alteração no enunciado	Excluído	-
56. Você já recebeu informações sobre alimentação saudável em um serviço de saúde, na escola da sua comunidade ou em outro serviço, por exemplo, de assistência social?	1,00	1,00	1,00	Sem alteração	Alteração no enunciado	Excluído	-
INCENTIVO À CULINÁRIA						
57. Você já participou de uma vivência culinária ou de atividade em grupo incentivando a cozinhar em casa, realizada em um serviço de saúde, na escola da sua comunidade ou em outro serviço, por exemplo, de assistência social?	1,00	1,00	1,00	Pequena alteração no enunciado	Alteração no enunciado	Excluído	-
INCENTIVO À AGRICULTURA FAMILIAR E URBANA DE BASE AGROECOLÓGICA						
58. Você consegue comprar em locais que vendem alimentos produzidos por agricultores familiares, por exemplo: feira, galpão, lojas, sítios...?	0,89	0,89	1,00	Alteração no enunciado	Alteração no enunciado	Excluído	-
GARANTIA DO DIREITO HUMANO À ALIMENTAÇÃO ADEQUADA E SAUDÁVEL						
59. Você consegue realizar, pelo menos, três refeições, todo dia sem ter que deixar de pagar outra despesa da casa, por exemplo, água e luz?	1,00	1,00	1,00	Sem alteração	Excluído	-	-
GARANTIA DA SAÚDE						
60. Você conseguiu ser atendida no serviço público de saúde quando procurou?	0,90	0,90	1,00	Sem alteração	Sem alteração	Excluído	-
61. Você já recebeu um atendimento de pior qualidade no serviço de saúde por causa da cor da sua pele?	0,90	0,89	1,00	Sem alteração	Sem alteração	Excluído	-
GARANTIA DA EDUCAÇÃO						
62. Você precisou parar de estudar para trabalhar?	0,90	0,90	0,90	Sem alteração	Alteração no enunciado	Excluído	-

C: clareza; CVR: *content validity ratio*; P: pertinência; R: relevância.

Fonte: elaboração própria.

* CVR = (número de especialistas que classificaram cada item como “relevante” [4 ou 3, onde 4 = “altamente relevante”; 3 = “bastante relevante” ou “altamente relevante”, mas precisa ser reescrito] - número total de respondentes/2)/número total de respondentes/2. O mesmo se aplica para “pertinente” e “claro”.

### Pré-teste I

A primeira jornada de pré-teste foi realizada com 10 mulheres com idade entre 33 e 58 anos, com filhos, renda domiciliar entre R$ 500,00 ou menos e R$ 3.000,00, e nível de escolaridade entre Ensino Fundamental completo e Médio completo.

Quanto à operacionalização do protótipo, observou-se: fácil aplicação; adesão das entrevistadas; e algum impacto emocional nos itens referentes ao acesso a direitos como alimentação, proteção social, sistema de saúde, educação e direitos trabalhistas. A duração média das entrevistas foi de 37 minutos.

Quanto à redação dos itens, no bloco de caracterização, que abrange 11 questões, foram sugeridos aprimoramento da clareza e da objetividade dos enunciados; aprimoramento das opções de resposta, alteração do termo “trabalho irregular” por “trabalho temporário ou sem horário fixo” e reordenamento de algumas questões.

### Oficina com especialista em desenvolvimento de instrumentos

A oficina com especialista em desenvolvimento de instrumentos trouxe contribuições fundamentais para qualificar o processo de desenvolvimento do protótipo. Como principais resultados dessa etapa, podemos apontar:

(a) decisão sobre não incluir todos os componentes do MCAC na elaboração de itens, sendo mantidos todos os componentes relativos aos níveis do Agente e do Microssistema, além do componente “disponibilidade e acesso a alimentos *in natura* ou minimamente processados” referente ao Mesossistema, por estarem diretamente relacionados ao construto de autonomia culinária (Quadros 2 e 3); foram excluídos os componentes relativos ao Exossistema e ao Macrossistema (14 itens) por informarem sobre questões mais amplas, como a presença de políticas públicas para garantia de segurança alimentar e nutricional, que tem influência sobre o construto de autonomia culinária, mas não é específica dele (Quadro 4);

(b) revisão das dimensões individual, relacional e comunitária e de políticas públicas, inicialmente propostas por abrangerem diferentes aspectos do construto, o que provavelmente não seria confirmado na etapa de validação dimensional, optando-se, então, por manter os próprios componentes do MCAC como dimensões conjecturadas até que análises psicométricas possam fornecer informações que orientem a decisão final;

(c) posicionamento dos itens em um gradiente de intensidade de manifestação do construto, definido por níveis inicial, intermediário e elevado de autonomia culinária, iniciando o desenho de um mapa de construto. Esse processo indicou que alguns itens estavam englobando diferentes níveis de manifestação do construto de autonomia culinária, apresentando, portanto, menor capacidade de discriminação dos indivíduos;

(d) revisão do conteúdo e da semântica dos itens para superar a sobreposição de conteúdos, o uso de termos pouco diretivos e a enunciação de exemplos, que poderiam causar confusão e dificuldade de interpretação da respondente, levando a respostas inconsistentes;

(e) revisão de alguns enunciados a fim de excluir as opções de resposta “não sabe”, “não quis responder” e “mora sozinha”, que culminou com a definição de um bloco específico, composto por três itens, para pessoas que não moram sozinhas;

(f) decisão por não incluir a formulação de um escore na etapa prototípica, considerando que a atribuição de valores absolutos às opções de resposta poderia não corresponder à estrutura dimensional conjeturada; e dados sobre confiabilidade.

As alterações relativas aos itens geraram o desdobramento de alguns deles em até quatro novos itens, totalizando 64 itens na segunda versão do protótipo.

### Pré-teste II

A segunda jornada de pré-teste foi realizada com dois grupos de mulheres. O primeiro contou com 10 mulheres socialmente vulneráveis com idade entre 27 e 70 anos; com filhos (duas com netos), renda entre R$ 501,00 e R$ 3.000,00, e nível de escolaridade entre sem estudo e Ensino Médio completo. Quanto à raça/cor, cinco se autodeclararam pretas, três pardas e duas, brancas. O segundo foi composto por 10 mulheres de classe média com idade entre 28 e 51 anos, com filhos, renda entre R$ 5.001,00 e R$ 10.000,00 ou mais e com Ensino Superior completo. Quanto à raça/cor, oito se autodeclararam brancas e duas, pardas.

Quanto à operacionalização, as entrevistas duraram, em média, 23 e 31 minutos, respectivamente. As entrevistadas demonstraram interesse em participar e em responder todo o questionário, sem resistência quanto a qualquer item.

Quanto aos itens, foram registradas: sugestões para aprimorar o enunciado de 13 itens; confirmação da necessidade de utilizar frequência de tempo como opção de resposta para três itens; facilidade de compreensão de termos como “pratos”, “textura”, “espaço suficiente”, “legumes”, “verduras” e “alimentos plantados em casa”, sendo este último uma sugestão de entrevistada para substituir o termo “horta”; dificuldade de compreensão dos termos “fazer adaptações em receitas” e “agrotóxicos”. Com base nesses achados, foram excluídos três itens por falta de clareza (sobre lista de compras, resíduos de embalagens e compra direta de produtores); desdobrados quatro itens em oito (sobre pré-preparo e a compra de alimentos); e substituído na íntegra o item sobre confiança em utilizar receitas porque as entrevistadas informaram compreensão contrária ao nele proposto.

Destaca-se como principal questão a experimentação de opções de resposta sobre a própria pessoa e sobre outras pessoas da casa que também fazem compras de alimentos. Essas opções foram utilizadas em seis itens relativos ao componente “poder de compra com análise crítica”. Observou-se relativa facilidade de resposta uma vez que a entrevistada conhecia em profundidade a dinâmica do domicílio.

### Versão final do protótipo

Do total de 62 itens, ao longo das etapas da fase prototípica, a maior parte deles sofreu algum grau de alteração de enunciado ou opção de resposta, alguns foram excluídos provisoriamente e poucos foram excluídos permanentemente ou não sofreram nenhuma alteração (Quadros 2, 3 e 4).

A terceira versão (considerada final) do protótipo do instrumento abrange três níveis do MCAC que abarcam 12 dimensões (Quadro 5). O questionário produzido apresenta, em sua parte inicial, um bloco com 10 itens sobre a caracterização sociodemográfica da pessoa, seguido do IAC, composto por: um bloco sobre conhecimentos, hábitos e atitudes individuais com 38 itens; e um bloco sobre aspectos do domicílio e práticas dos moradores, com 26 itens, sendo que três deles são aplicáveis somente a pessoas que moram com, pelo menos, mais um adulto ou criança maior de 10 anos. O protótipo apresenta cinco diferentes estruturas de opções de resposta. O IAC pode ser acessado na íntegra em: https://bit.ly/questionárioIAC.

**Quadro 5:** Enunciado e respectivas opções de resposta dos itens que compõem a versão final do protótipo segundo níveis do modelo conceitual de autonomia culinária e respectivas dimensões. Brasil, 2021.

ENUNCIADO DO ITEM E OPÇÃO DE RESPOSTA	
NÍVEL AGENTE	DIMENSÃO - CONHECIMENTO EM ALIMENTAÇÃO E NUTRIÇÃO
	1. Vou ler uma lista de alimentos e você vai me dizer se, na sua opinião, ele faz parte de uma alimentação saudável no dia a dia. Arroz? (C35.1) Biscoito recheado/bolacha recheada? (C35.2) Feijão? (C35.3) Frutas? (C35.4) Legumes? (C35.5) Macarrão instantâneo (tipo Miojo)? (C35.6) Refrigerante? (C35.7) Salsicha? (C35.8) Tempero pronto (tipo Sazón)? (C35.9) Verduras? (C35.10) ( ) Não ( ) Sim
	2. Na sua opinião, usar diferentes legumes ou verduras para preparar as refeições da semana faz parte de uma alimentação saudável? (C36) ( ) Não ( ) Sim
	3. Na sua opinião, o congelamento é uma forma de fazer os alimentos durarem mais tempo, sem estragar? (C37) ( ) Não ( ) Sim
	4. Na sua opinião, a comida que sobrou na panela e que vai ser aproveitada em outra refeição deve sempre ser guardada na geladeira? (C38) ( ) Não ( ) Sim
	DIMENSÃO - INTERESSE PARA COZINHAR
	5. Você tem satisfação em preparar pratos diferentes da comida do dia a dia? (C12) ( ) Não ( ) Sim
	6. Você tem satisfação em preparar a comida do dia a dia? (C13) ( ) Não ( ) Sim
	7. Você tem satisfação em preparar o mesmo alimento de formas diferentes? (C14) ( ) Não ( ) Sim
	DIMENSÃO - CONFIANÇA PARA COZINHAR
	8. Você se sente confiante para preparar a comida do dia a dia sem seguir receita? (C32) ( ) Não ( ) Sim, às vezes ( ) Sim, quase sempre ( ) Sim, sempre
	9. Você se sente confiante para preparar um prato diferente seguindo uma receita? (C33) ( ) Não ( ) Sim, às vezes ( ) Sim, quase sempre ( ) Sim, sempre
	10. Você se sente confiante para preparar de novo uma receita que tenha dado errado antes? (C34) ( ) Não ( ) Sim, às vezes ( ) Sim, quase sempre ( ) Sim, sempre
	DIMENSÃO - DISPOSIÇÃO PARA COZINHAR
	11. Você tem ânimo para cozinhar todo dia? (C15) ( ) Não ( ) Sim
	12. Você tem ânimo para cozinhar em alguns dias da semana? (C16) ( ) Não ( ) Sim
	13. Você tem ânimo para cozinhar em ocasiões especiais? (C17) ( ) Não ( ) Sim
	DIMENSÃO - HABILIDADES CULINÁRIAS
	14. Você se sente capaz de preparar arroz simples? (C04) ( ) Não ( ) Sim
	15. Você se sente capaz de preparar feijão simples? (C05) ( ) Não ( ) Sim
	16. Você se sente capaz de limpar verduras para preparar uma salada? (C06) ( ) Não ( ) Sim
	17. Você se sente capaz de cortar legumes em mais de um formato? Por exemplo, cortar em rodelas, ralar, cortar em cubos ou em qualquer outro formato. (C07) ( ) Não ( ) Sim
	18. Você se sente capaz de temperar os alimentos utilizando somente temperos naturais? (C08) ( ) Não ( ) Sim
	19. Você costuma se organizar para ter em casa os alimentos básicos para cozinhar? (C18) ( ) Não ( ) Sim
	20. Você costuma planejar, com antecedência, o que vai cozinhar ao longo da semana? (C19) ( ) Não ( ) Sim
	21. Você costuma criar pratos com os alimentos disponíveis em casa? (C20) ( ) Não ( ) Sim
	22. Você costuma fazer mudanças nos ingredientes ou na forma de preparo quando prepara um prato? (C21) ( ) Não ( ) Sim
	23. Você costuma provar a comida enquanto está cozinhando? (C22) ( ) Não ( ) Sim
	24. Você costuma cheirar a comida enquanto está cozinhando? (C23) ( ) Não ( ) Sim
	25. Você costuma avaliar a textura da comida para saber se está pronta? (C24) ( ) Não ( ) Sim
	26. Você costuma sentir o cheiro da comida para saber se está pronta? (C25) ( ) Não ( ) Sim
	DIMENSÃO - ATITUDE ESTRATÉGICA
	27. Para adiantar o preparo das refeições, você costuma deixar na geladeira (C26): Temperos já descascados ou picados? (C26.1) Carnes em geral temperadas? (C26.2) Verduras já lavadas? (C26.3) ( ) Não ( ) Sim
	28. Você costuma deixar feijão de molho? (C27) ( ) Não ( ) Sim, às vezes ( ) Sim, quase sempre ( ) Sim, sempre
	29. Você deixa feijão cozido para mais de uma refeição? (C28) ( ) Não ( ) Sim, às vezes ( ) Sim, quase sempre ( ) Sim, sempre
	30. Você deixa comida pronta para mais de uma refeição? (C29) ( ) Não ( ) Sim, às vezes ( ) Sim, quase sempre ( ) Sim, sempre
	31. Você consegue preparar mais de um alimento ao mesmo tempo quando está cozinhando? (C30) ( ) Não ( ) Sim, às vezes ( ) Sim, quase sempre ( ) Sim, sempre
	32. Você consegue dedicar tempo suficiente para cozinhar em casa a comida do dia a dia? (C31) ( ) Não ( ) Sim, às vezes ( ) Sim, quase sempre ( ) Sim, sempre
	DIMENSÃO - EXPERIÊNCIAS E VIVÊNCIAS EM CULINÁRIA
	33. Você aprendeu a cozinhar com pessoas da sua família? (C01) ( ) Não ( ) Sim
	34. Você tem boas lembranças de cozinhar com familiares em casa? (C02) ( ) Não ( ) Sim
	35. Você já buscou ajuda de familiares quando teve dúvidas para cozinhar? (C03) ( ) Não ( ) Sim
	36. Você se sente capaz de preparar mais de um tipo de prato com arroz? (C09) ( ) Não ( ) Sim
	37. Você se sente capaz de preparar mais de um tipo de prato com legumes? (C10) ( ) Não ( ) Sim
	38. Você se sente capaz de preparar mais de um tipo de prato com carnes em geral? (C11) ( ) Não ( ) Sim
NÍVEL MICROSSISTEMA	DIMENSÃO - COZINHA COM INFRAESTRUTURA BÁSICA
	39. Na sua casa, tem espaço suficiente para preparar as refeições do dia a dia? (D01) ( ) Não ( ) Sim
	40. Na sua casa, tem espaço suficiente para guardar alimentos? (D02) ( ) Não ( ) Sim
	41. Na sua casa, tem fogão funcionando? (D03) ( ) Não ( ) Sim, mas não funciona bem ( ) Sim, funciona bem
	42. Na sua casa, tem geladeira funcionando? (D04) ( ) Não ( ) Sim, mas não funciona bem ( ) Sim, funciona bem
	43. Na sua casa, tem água encanada na cozinha? (D05) ( ) Não ( ) Sim, às vezes ( ) Sim, quase sempre ( ) Sim, sempre
	44. Na sua casa, tem gás de cozinha? (D06) ( ) Não ( ) Sim, às vezes ( ) Sim, quase sempre ( ) Sim, sempre
	45. Na sua casa, tem luz na cozinha? (D07) ( ) Não ( ) Sim, às vezes ( ) Sim, quase sempre ( ) Sim, sempre
	DIMENSÃO - IMPORTÂNCIA ATRIBUÍDA À CULINÁRIA
	46. Na sua casa, as pessoas costumam fazer pelo menos uma refeição por dia juntas? (D24) ( ) Não ( ) Sim, algumas pessoas ( ) Sim, todas as pessoas
	47. Sem contar você, na sua casa, as pessoas gostam de cozinhar em casa? (D25) ( ) Não ( ) Sim, algumas pessoas ( ) Sim, todas as pessoas
	48. Sem contar você, na sua casa, as pessoas gostam de preparar as receitas tradicionais da família? (D26) ( ) Não ( ) Sim, algumas pessoas ( ) Sim, todas as pessoas
	DIMENSÃO - COMPARTILHAMENTO DAS ATIVIDADES CULINÁRIAS
	49. Na sua casa, quem costuma cozinhar no dia-a-dia? (D08) ( ) Só você ( ) Somente outra(s) pessoa(s) ( ) Você e outra(s) pessoa(s)
	50. Na sua casa, quem costuma lavar a louça? (D09) ( ) Só você ( ) Somente outra(s) pessoa(s) ( ) Você e outra(s) pessoa(s)
	51. Na sua casa, quem costuma fazer a limpeza da casa toda? (D10) ( ) Só você ( ) Somente outra(s) pessoa(s) ( ) Você e outra(s) pessoa(s)
	52. Na sua casa, quem costuma fazer as compras de alimentos? (D11) ( ) Só você ( ) Somente outra(s) pessoa(s) ( ) Você e outra(s) pessoa(s)
	53. Na sua casa, quem costuma decidir quais alimentos serão comprados? (D12) ( ) Só você ( ) Somente outra(s) pessoa(s) ( ) Você e outra(s) pessoa(s)
	DIMENSÃO - PODER DE COMPRA COM ANÁLISE CRÍTICA
	54. Você ou outro morador da sua casa evita comprar algum alimento porque faz mal à saúde? (D13) ( ) Não ( ) Sim, só você ( ) Sim, somente outra(s) pessoa(s) ( ) Sim, você e outra(s) pessoa(s)
	55. Você ou outro morador da sua casa evita comprar algum alimento porque é produzido com agrotóxico ou pesticida? (D14) ( ) Não ( ) Sim, só você ( ) Sim, somente outra(s) pessoa(s) ( ) Sim, você e outra(s) pessoa(s)
	56. Quando escolhe alimentos para comprar, você ou outro morador da sua casa costuma comparar os preços de alimentos? (D15) ( ) Não ( ) Sim, só você ( ) Sim, somente outra(s) pessoa(s) ( ) Sim, você e outra(s) pessoa(s)
	57. Quando escolhe alimentos para comprar, você ou outro morador da sua casa costuma conferir no rótulo:(D16) Prazo de validade? (D16.1) Lista de ingredientes? (D16.2) Tabela de informação nutricional? (D16.3) ( ) Não ( ) Sim, só você ( ) Sim, somente outra(s) pessoa(s) ( ) Sim, você e outra(s) pessoa(s)
NÍVEL MESOSSISTEMA	**DIMENSÃO - DISPONIBILIDADE E ACESSO A ALIMENTOS *IN NATURA* OU MINIMAMENTE PROCESSADOS**
	58. Você ou outros moradores da sua casa costumam utilizar alimentos plantados em casa nas refeições? (D17) ( ) Não ( ) Sim
	59. É fácil comprar verduras, com bom preço, para sua casa? (D18) ( ) Não ( ) Sim
	60. É fácil comprar verduras, de boa qualidade, para sua casa? (D19) ( ) Não ( ) Sim
	61. É fácil comprar verduras, com variedade, para sua casa? (D20) ( ) Não ( ) Sim
	62. É fácil comprar frutas, com bom preço, para sua casa? (D21) ( ) Não ( ) Sim
	63. É fácil comprar frutas, de boa qualidade, para sua casa? (D22) ( ) Não ( ) Sim
	64. É fácil comprar frutas, com variedade, para sua casa? (D23) ( ) Não ( ) Sim

Fonte: elaboração própria.

Nota: código alfanumérico entre parênteses significa a posição do item no questionário.

## Discussão

O processo inicial de construção do instrumento buscou acompanhar a amplitude do MCAC por meio da definição de categorias teóricas e assertivas e, por fim, itens sobre condições e oportunidades objetivas para desenvolver autonomia culinária para todos os componentes desse modelo conceitual. Um estudo de validação da *Cooking and Food Provisioning Action Scale* (CAFPAS; Escala de Ação de Cocção e Fornecimento de Alimentos), instrumento que propõe articular práticas individuais ao contexto, apontou como limitação dessa escala a incorporação somente do tempo disponível para cozinhar como elemento contextual, indicando a necessidade de inclusão de itens que captem outros elementos ^23^.

No presente estudo, itens relativos aos níveis do Exossistema e do Macrossistema também não foram validados para inclusão na versão final do protótipo.

Os componentes presentes no Exossistema apontam para a necessidade de fortalecer a liberdade e a autonomia das pessoas e, consequentemente, diminuir sua vulnerabilidade social por meio de políticas públicas e de um modelo de desenvolvimento que contribua para a superação das desigualdades sociais. Sendo assim, a aplicação do IAC pode ser potencializada pela aplicação concomitante de escalas sobre uso saudável do tempo, desigualdade racial, desigualdade de gênero, desigualdade econômica e (in)segurança alimentar e nutricional, com destaque para a *Escala Brasileira de Insegurança Alimentar* ^24^.

Um inquérito representativo da população norte-americana ^9^ realizado em 2015 com o propósito de examinar como as barreiras de acesso à alimentação saudável estão associadas a comportamentos alimentares e à culinária doméstica segundo renda domiciliar apontou que extratos de baixa renda estiveram mais associados a todos os tipos de barreiras citados no estudo: preço; tempo para compra; qualidade de itens disponíveis para compra; seleção de itens disponíveis para compra; distância até o estabelecimento usual de compra; horário de funcionamento dos estabelecimentos; dificuldades físicas; e falta de transporte. Já em outro estudo ^23^, percebeu-se associação entre maior frequência de preparo de refeições em casa e qualidade da alimentação entre adultos, tanto de baixa quanto de alta renda, sendo a associação ainda mais forte em adultos de alta renda.

Considera-se que elementos dos componentes do Macrossistema, como direito à saúde, à educação e à alimentação adequada e saudável, e dos componentes do Cronossistema, como a valorização da culinária e de sistemas alimentares mais saudáveis e sustentáveis, poderiam ser contemplados em itens já incluídos ou a serem incluídos: (a) na caracterização sociodemográfica; (b) por meio de módulo(s) complementar(es) ao IAC com foco na avaliação de políticas públicas; e/ou (c) por meio da estratificação de diferentes grupos populacionais em etapas posteriores de desenvolvimento do instrumento, como a avaliação da validade de construto externa.

Quanto à estrutura dos itens que foram mantidos na versão final do protótipo, aqueles que buscam aferir conhecimentos apresentam relação com os instrumentos desenvolvidos para avaliar *food literacy* ^3^. No entanto, em função da forte influência da classificação Nova ^25^ e do *Guia Alimentar para a População Brasileira* ^26^ sobre o MCAC, os itens propostos para o IAC incorporaram elementos sobre os grupos de alimentos definidos em função da extensão e do propósito de processamento. De acordo com o conceito de autonomia culinária, os alimentos *in natura* ou minimamente processados deveriam ser a base das refeições diárias, complementadas com alimentos processados, evitando-se o uso de alimentos ultraprocessados ^26^. É essa perspectiva de culinária doméstica que orientou a elaboração desse protótipo, em oposição a uma concepção mais permissiva, que também considera como culinária doméstica, por exemplo, a finalização de preparações culinárias industrializadas ^27^.

Os itens selecionados para medir as dimensões de habilidades culinárias e confiança em cozinhar tomaram como base as escalas de *cooking skills* e *food skills* ^5^, mas se buscou valorizar menos o domínio de técnicas culinárias e mais o preparo da comida presente no cotidiano da cultura alimentar brasileira. Essa perspectiva foi intensificada nos itens relativos às experiências e vivências culinárias, considerando que a prática da culinária na realidade brasileira é marcada por um aprendizado que é muito mais observado, vivido e compartilhado do que estudado e lido. Essa concepção se apoia na categoria teórica da oralidade, que expressa outras formas de aprendizado do povo negro escravizado e de seus descendentes privados de acesso ao ensino formal e vem sendo explorada em narrativas de decolonialidade do conhecimento ^28^. Outro estudo, ao considerar o ato de cozinhar como prática social, aponta a força de elementos não cognitivos, como materiais, competências e significados, na complexa rede de relações que circundam o cozinhar por populações em situação de insegurança alimentar e que sustentam a capacidade desses grupos para cozinhar mesmo diante de muitas limitações ^29^.

Grande parte dos itens selecionados para as dimensões que compõem o nível do Agente é fortemente inspirada no instrumento CAFPAS ^30^, desenvolvido com base no construto de *food agency*. Considera-se que a principal inovação do protótipo aqui apresentado é incorporar às práticas e conhecimentos individuais aspectos relativos ao ambiente físico do domicílio e ao ambiente relacional de seus moradores, que partilham a comida ali produzida, presentes nos itens relativos às dimensões que integram os níveis do Microssistema e do Mesossistema.

Os itens relativos à cozinha com infraestrutura básica e à disponibilidade de alimentos *in natura* ou minimamente processados no entorno do domicílio visam captar aspectos físicos que podem se apresentar como barreiras ou facilitadores para a autonomia culinária. Nesse instrumento, considerou-se como infraestrutura básica o espaço físico suficiente para armazenar alimentos e para cozinhar; e o acesso a insumos (água, luz e gás) e equipamentos (geladeira e fogão). Um estudo sobre domicílios com crianças em situação de insegurança alimentar observou um número menor de equipamentos para cozinhar, mas uma frequência de uso de utensílios e equipamentos semelhante a domicílios em situação de segurança alimentar. Os autores sugerem que novos estudos investiguem o impacto do maior ou menor número de equipamentos nos comportamentos de culinária ^31^.

Também compõe esse instrumento o abastecimento da casa com alimentos, em especial os não estocáveis, adquiridos a intervalos menores. A distância entre o domicílio e os estabelecimentos que oferecem alimentos de qualidade, variados e a preços acessíveis tem sido estudada como aspecto facilitador ou barreira para a compra de alimentos, em especial, frutas, legumes e verduras ^32^.

A questão relacional entre os moradores do domicílio, fortemente pautada em aspectos simbólicos sobre cozinhar no ambiente doméstico ^33^ ^,^ ^34^, também está contemplada no instrumento proposto. Alguns itens captam manifestações sobre a convivência com pessoas que atribuem importância à prática cotidiana da culinária, ao compartilhamento de tarefas relacionadas ao preparo das refeições e a uma postura crítica sobre os tipos de alimentos adquiridos para preparar as refeições. Algumas opções de resposta oferecidas pelo instrumento captam não só o posicionamento individual, mas o quanto essas práticas são compartilhadas com os demais moradores do domicílio. A importância dessas questões é corroborada em estudo qualitativo com abordagem sociopsicológica sobre as escolhas relacionadas à culinária que indicou que as escolhas das pessoas que cozinhavam eram fortemente baseadas em valores, independentemente dos desafios para a prática ^35^.

A pertinência de o instrumento contemplar aspectos relacionais referentes à desigualdade na divisão sexual do trabalho ^36^ é reforçada pelos resultados da *Pesquisa Nacional por Amostra de Domicílios Contínua*, que apontam que a maior taxa de realização de afazeres domésticos ocorreu entre as mulheres pretas (94,1%) e a menor, entre os homens pardos (76,5%). As tarefas de “preparar ou servir alimentos, arrumar a mesa ou lavar louça” foram realizadas por 95,5% das mulheres e por 62% dos homens ^17^.

Ainda em relação aos aspectos simbólicos, a prática da culinária doméstica vem sendo desvalorizada pela ação predatória das indústrias de alimentos via oferta e propaganda de alimentos ultraprocessados, ações altamente lucrativas, travestidas de promessas de praticidade, tempo livre para a família, liberdade e empoderamento feminino pautadas em modelos de desenvolvimento excludentes e desiguais ^37^.

Outro grupo de itens, relacionado ao poder de compra com análise crítica, visa captar a relação, estabelecida ou não, pela pessoa e outros moradores do domicílio, entre a prática da culinária doméstica e os sistemas alimentares. Esses itens estão em consonância com o que vem sendo recomendado por especialistas em nível mundial: transformações radicais nas cadeias de abastecimento de alimentos, nos ambientes alimentares e nas práticas dos consumidores, assim como um redirecionamento à produção global de alimentos a fim de garantir sistemas alimentares comprometidos com a saúde, a equidade, a sustentabilidade e a justiça social ^38^.

A principal contribuição deste estudo foi a construção do protótipo de um instrumento baseado em um modelo conceitual validado, estruturado para captar a complexidade da prática da culinária doméstica e coerente com a realidade brasileira. Os resultados de sua aplicação poderão qualificar as abordagens de iniciativas da sociedade civil e de políticas públicas brasileiras.

Considera-se como fortaleza do estudo a adoção de referencial teórico metodológico rigoroso, que exige a realização de diferentes etapas para o traslado do modelo teórico (no caso, MCAC) para o instrumento e a escuta sistemática de especialistas e da população de interesse.

Aponta-se como limitação a insuficiência das jornadas de pré-teste para viabilizar a quantidade necessária de entrevistas para a realização de testes preliminares de confiabilidade ^18^. Ressalva-se que parte das entrevistas foi realizada ainda durante a pandemia de COVID-19.

Cabe reconhecer que novos arranjos familiares e novas conformações do ambiente e do trabalho doméstico podem indicar a necessidade de estudos de adaptação desse instrumento e sua validação para diferentes identidades de gênero e orientações sexuais.

Dando sequência ao desenvolvimento do IAC, está em andamento um estudo de validação dimensional e de confiabilidade com mais de 1.000 mulheres trabalhadoras em três diferentes regiões brasileiras. A avaliação psicométrica sobre o desempenho do IAC também poderá confirmar a adequação do seu uso em estudos populacionais e na prática clínica. Por fim, a aplicação futura desse instrumento em estudos locais, regionais e nacionais poderá subsidiar a formulação e implementação de políticas públicas de alimentação e nutrição, assim como qualificar a atenção nutricional no Sistema Único de Saúde (SUS).
